# “They Protect us as if they were our Mom” Masking Attitudes from Freelist Survey Data and Qualitative Interviews in San Diego School Communities

**DOI:** 10.1007/s10900-023-01245-1

**Published:** 2023-06-14

**Authors:** V. Omaleki, A. Flores Gonzalez, A. Hassani, M. Flores, S. Streuli, A. Wishard Guerra, R. Fielding-Miller

**Affiliations:** 1 Herbert Wertheim School of Public Health, University of California, San Diego, La Jolla, CA United States of America; 2 Department of Education Studies, University of California, San Diego, La Jolla, CA United States of America

**Keywords:** Mask, COVID-19, Attitudes, Equity, Public Health, Mixed-Methods

## Abstract

Background: Despite being disproportionately impacted by COVID-19 due to a lack of structural support, marginalized communities have been largely ignored in the politically polarized debate over school masking. In response to this, we sought to explore masking attitudes by centering the voices of parents and children at historically marginalized, predominantly Hispanic schools in southern California. Methods: We conducted a mixed-methods study with parents and children attending 26 low-income predominantly Hispanic-serving elementary schools. A random sample of parents was asked to provide a freelist of words they associate with masking. A subset of parents with children aged 4–6 was recruited from these surveys to participate in parent-child interviews (PCI). We calculated Smith’s salience index for all unique items, stratifying by language (English/Spanish). Item salience guided PCI thematic analysis for additional context and meaning. Results: 648 participants provided 1118 unique freelist items in English and Spanish. 19 parent-child pairs were interviewed, 11 in Spanish and 8 in English. The most salient words were “safety”(0.37), “protection”(0.12), “prevention”(0.05), “health”(0.04), “good”(0.03), “can’t breathe”(0.03), “necessary”(0.02), “care”(0.02), “precaution”(0.02), and “unnecessary”(0.02). Spanish speakers had a more favorable view of masking than English speakers, particularly regarding “protection” (0.20 vs 0.08) and “prevention” (0.10 vs 0.02). Discussion: Masking is an affordable individual-level risk mitigation that protects the communities that have inequitably shouldered the burdens of the COVID-19 pandemic. We recommend that policymakers prioritize the views of those most impacted when deciding on risk mitigation policies like school masking.

## Background

Masking is an effective strategy to slow the transmission of airborne pathogens, including influenza, RSV [1, 2], and SARS-CoV-2 [3, 4]. The American Academy of Pediatrics strongly recommends the use of masking in school settings for individuals who are not fully vaccinated against COVID-19, have specific healthcare needs, or live in communities with currently high levels of virus transmission [5]. The first point is particularly salient in elementary school settings as only 30% of children ages 5–12 in the United States have accessed the full COVID-19 vaccination series, and children from economically marginalized communities are significantly less likely to be vaccinated [6]. The same trends can be seen in San Diego County [7].

Despite the fact that masking has been shown to be a low-cost, effective tool to reduce transmission in school settings [8], with no credible evidence suggesting it affects social [9], academic, or emotional development [10], the role of masking as a public health measure has engendered significant policy and cultural debate [11]. In particular, masking policies in school settings have created highly charged discussions around choice and public safety [12].

The perception of mask-wearing as a politically controversial issue has largely been shaped by privileged voices [13]. In the United States, white men are the most likely demographic to express negative attitudes towards masking and school masking policies are more likely to be male and white [11, 14]. These negative attitudes have been largely centered in political and media discourse, while marginalized communities that have borne the brunt of morbidity, mortality [15], and economic pandemic effects [16–18] have been largely surpassed in the politically polarized debate over school masking [13].

We wanted to understand attitudes towards masking and masking policy in school communities. Our team studied parents’ and children’s experiences and attitudes toward masking at school during the pandemic after all California state mandates, San Diego County mandates, and district and individual school masking policies had been lifted. We sought to center the voices of those most impacted by the COVID-19 pandemic, parents and children at historically marginalized, predominantly Hispanic schools in southern California.

## Methods

The data for this study are part of a larger SARS-CoV-2 environmental monitoring intervention, the Safer at School Early Alert (SASEA) project [19]. The component reported here used a mixed-methods sequential explanatory design [20, 21] with freelisting activities embedded in a randomly distributed community survey followed by structured in-depth interviews. We chose freelisting because we wanted more nuance than just good/bad dichotomies. Freelisting is an excellent way to understand the meaning and boundaries of a cognitive domain - a mental category for a topic [21–25]. It is commonly used in ethnographic approaches when there is no known answer key [26]. Freelisting accesses cognitive domains from an emic (insider) perspective, without imposing the researcher’s perspective. [26]. Data were collected between October 2021 and June 2022 at 26 primary schools in central and southern San Diego County.

### Participants

Parents of children affiliated with any SASEA school site were eligible to participate in the study. SASEA school sites were located in low socioeconomic status communities with a high risk of COVID-19 transmission [19]. The school sites were divided into 4 clusters across 4 school districts, with the largest district divided into two clusters to account for the fact that one group of schools primarily served military-affiliated families, while the second cluster served a diverse mid-city neighborhood with many resettled refugee families.

Survey participants were recruited via stratified random sampling: We randomly selected 3 classrooms per school over the course of six survey waves. Every child in the selected classroom was given a paper flyer with survey information in English and Spanish. Teachers and principals were also encouraged to send home digital versions of the same flyer. The survey was self-administered via a web link. At the end of the survey, respondents who indicated that their child was between the ages of 4 and 6 were asked if they were interested in participating in a longer semi-structured interview.

### Survey Methods

Self-administered online surveys took 20 min to complete on average and contained items related to testing, vaccination, and masking behaviors along with parent and child demographics and perceptions of COVID-19 risk. The survey also included a prompt to freelist words that participants associated with masking (“What words come to mind when you think about masking in schools? List as many words as you like”).

### Parent-child Interview Methods

Eligible survey participants who indicated that they were interested in participating in an interview were contacted by a member of the study team, who explained the interview structure and scheduled a time if the participant was still interested. We conducted in-person interviews at their child’s school or a local park of their choice. Interviews were conducted in English and Spanish based on parent and child preferences. AF and another Spanish-English bilingual researcher conducted Spanish and English language interviews. VO, AH, and two other researchers conducted interviews in English.

Interviews followed a semi-structured format consisting of parent/interviewer, parent/child, and child/interviewer sections. First, interviewers asked parents about their and their child’s experiences of schooling during the pandemic. Then, the parent was instructed to ask their child about their understanding of COVID-19 and risk mitigation strategies. Finally, the interviewer asked the child about their perspectives and experiences of returning to in-person schooling and COVID-19 risk mitigation strategies, including masking. The interviewer took notes throughout the discussion, and all interviews were digitally recorded, transcribed verbatim, and translated as necessary.

### Analysis

The community survey freelisting data were cleaned and translated into English by AF and another Spanish-English bilingual researcher. We first created a scree plot of term frequency across all lists and then calculated item salience and the Smith’s S score for items across the whole sample and by respondent language (English and Spanish). Salience is calculated using the equation [salience = R x (1/(L-1))]. R is the order (rank) in which an item was named on an individual respondent’s list and L is the total length of the respondent’s list. Smith’s S is an indicator of salience across a group or sample in which the sum of salience scores for an item across a full sample is divided by the number of respondents in the sample, i.e., [Smith’s S = $\Sigma s \div N$ ] [27]. We calculated Smith’s S for the full sample, and then for Spanish and English-speaking respondents separately. Freelist data were cleaned in Excel and analyzed using the AnthroTools package in R [27].

We transcribed all PCIs verbatim with at least 2 researchers per document and translated Spanish interviews to English so that Spanish transcriptions and translations were side-by-side in the same document. Each transcription had two coders who used Dedoose [28]. We developed the initial codebook based on highly salient words identified in the freelisting exercise. After coding an initial subset of transcripts, we then refined this codebook based on emergent subthemes and contextual information related to the freelisting terms. Our team met weekly to discuss codebook development and coding progress, and all differences were resolved via consensus discussion.

### Ethics

This study was reviewed and approved by the University of California San Diego Institutional Review Board (UCSD IRB). Survey participants provided informed consent through REDCap before starting the survey [29, 30]. All survey participants were entered into a raffle to win one of the $100 gift cards per survey wave per regional cluster.

PCI interviews were held in outdoor settings to reduce the possibility of COVID-19 exposure and interviewers wore masks. PCI facilitators informed participants of the purpose of the study, and their rights, and obtained participants’ consent to record the interview via audio recorder. Participants were reminded that they were able to disengage or withdraw from the PCI at any time. PCI participants were compensated for their time with a $50 gift card ($25 for the parent and $25 for the child).

We shared preliminary study results in lay-friendly bilingual infographics on a community website which we heavily publicized in both English and Spanish [31].

## Results

648 participants completed the survey freelist question, 35.8% (N = 232) were Spanish speakers and 84.9% (N = 550) were women. We conducted 19 concurrent parent-child interviews, 11 in Spanish and 8 in English. 18 parents identified as female and 1 as male. Findings below are organized into themes found through freelisting and item salience followed by PCI quotations that give context to the themes.

### Freelisting and item Salience

**Fig. 1:**
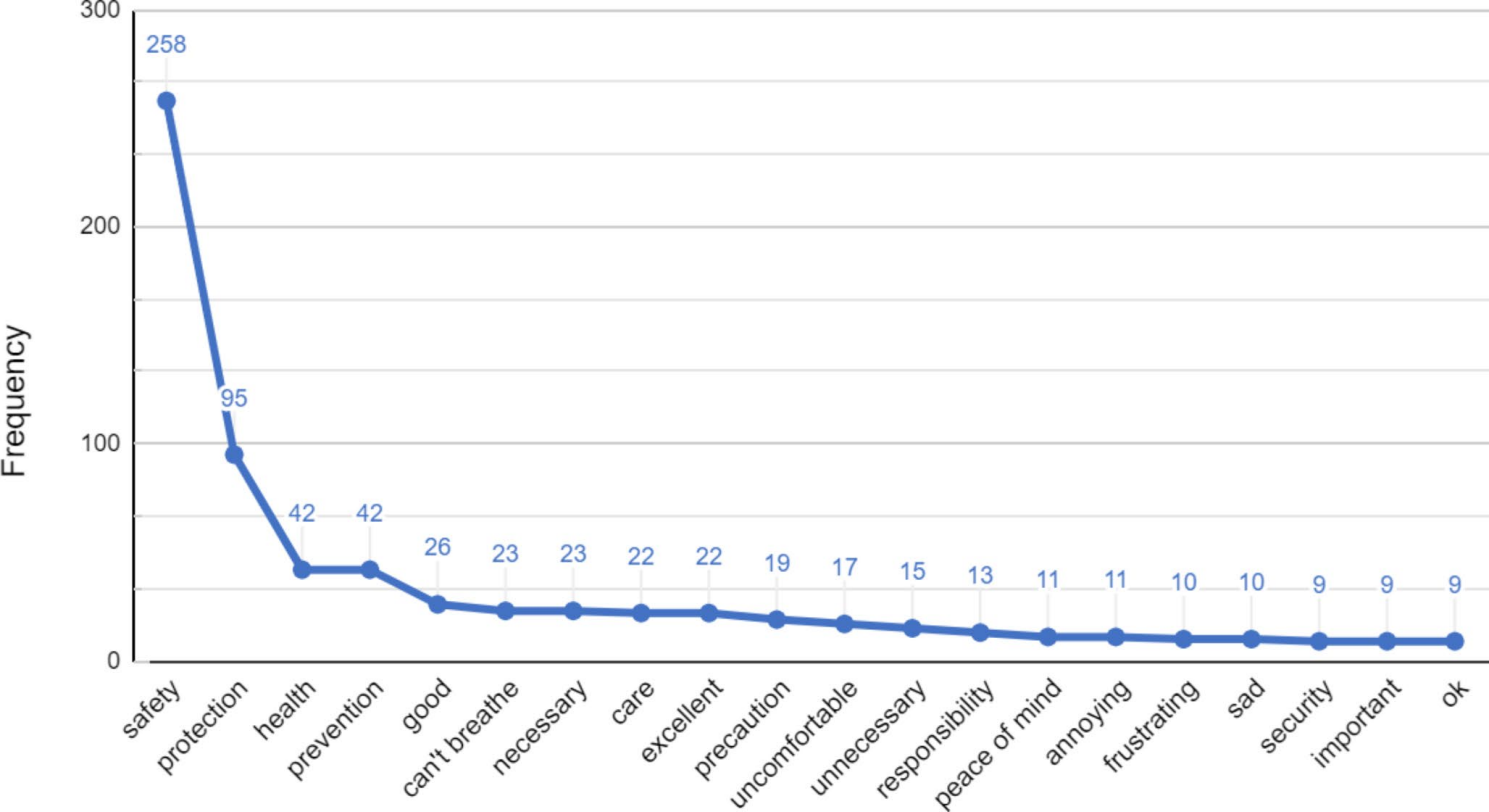
Overall Item Frequency for Freelist Associations to Masking

Participants listed 1118 unique items, and the average number of items provided was 1.73. Item Smith’s S values across the full sample are shown in Fig. 1. The most salient terms associated with masking were “safety” (0.37), “protection” (0.12), “prevention” (0.05), “health” (0.04), “good” (0.03), “can’t breathe” (0.03), “necessary” (0.02), “care” (0.02), “precaution” (0.02), and “unnecessary” (0.02).

**Fig. 2:**
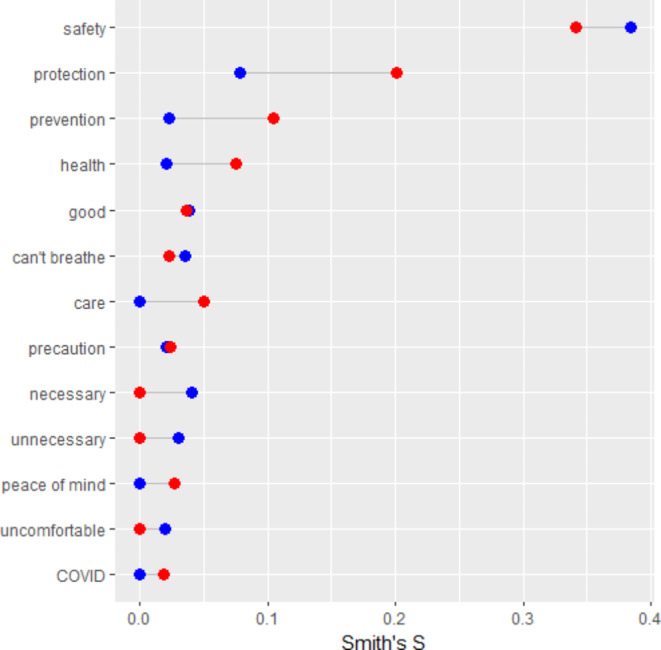
Masking Frequency: English (blue) v Spanish (red)

Although there is no formal statistical test for significant differences in mean Smith’s S index, we did identify trend differences for priorities between respondents who completed the survey in Spanish and English (Fig. 2). While “safety” was the most salient term for both Spanish and English respondents, protection, prevention, health, and care appeared to be more salient to respondents who completed the survey in Spanish than those who completed the survey in English.

### Safety

Safety was the most salient word overall and among both Spanish and English-speaking participants (0.34 vs. 0.38).

In the interviews, parents generally viewed masking as a “blanket” safety measure against COVID-19, though as one parent explains, not foolproof.

*Madre: No sé. O sea, no es que [las reglas] no me hagan sentir 100% segura. O sea, realmente si reduce mucho el, el contagio, pero yo pienso que sí siempre va a haber como un margen de… de que uno no va a saber que está enfermo o no lo tiene por no tener síntomas o algo. En algún momento, no sé, si come o no sé. Realmente no sé cómo son realmente en el salón. Pero si puede pasar con un niño de que se quite la máscara. Incluso se quitan las máscaras cuando comen.*

*Parent: I don’t know. like, it’s not that they [the rules] don’t make me feel 100% safe. Like, really it does reduce quite a lot the, the spread, but I think that yes there is always going to be like a margin of… that one is not going to know that they are sick, or they do not have it because they do not have symptoms or something. At some point, I don’t know, if they eat or I don’t know. Really, I don’t know how they really are in the classroom. But it can happen with a kid that they take off the facemask. They even take off the facemask when they eat.*

#### Spanish-speaking Parent, Age 31

*Parent: It was a very big release for me, not only that the mask wearing that it was mandated that they all wore masks…So that was something like I said that, that the school had reassured us that they were gonna work to the best of their ability, to make sure that we were all gonna be safe - That the kids are all gonna be safe.*

### English-speaking Parent, Age 42

#### Protection

Protection was the second most salient word across the three groups. There was a notable difference in Spanish speakers with a higher Smith’s S (0.2) compared to English speakers (0.08).

Some participants described masking as an individual protection factor in the case that preventative measures fail.

### Madre: ¿El uso de cubrebocas? … Y ¿el uso de desinfectante?

#### Parent: The use of Facemasks? …. And the use of Sanitizer?

*Niña: Si, y también uhm… que nos protegen.*

*Child: Yes, and also uhm… that they protect us.*

### Madre: ¿Oh sí? Y ¿Cómo los Protegen?

#### Parent: Oh yeah? And how do they Protect you?

*Niña: Uh, nos protegen como si fuera como nuestra mamá.*

*Child: Uh, they protect us as if they were our mom.*

### Spanish-speaking Parent, Age 44 and Spanish-speaking Child, Age 6

*Madre: Entonces este, “se [miembro de familia que falleció] contagió.” Y le, le explicamos [a la niña], “eh, ¿Por qué crees que se haya- ¿Por qué crees que se murió?” “Por el COVID, dice me dijeron, me dijo mi madrina que por COVID. Aha. Y, y pero que también a él no le gustaba usar su mascara.” Entonces le decimos “y, y ¿tu crees que tienes que usarla?” Dice, “Sí. Porque” dice “yo no me quiero morir.” [tono humorístico del padre]*

*Parent: Then well, “he [deceased family member] was infected” And we, we explained to her [Child], “eh, why do you believe he- why do you believe he died?” “Because of COVID, they say I was told, my godmother told me that because of COVID. Aha. And, and but also that he did not like to wear a facemask.” So, we tell her “And, and do you believe that you have to use it?” She says, “yes, because” she says, “I do not want to die.” [humorous tone from parent]*

### Spanish-speaking Parent, Age 32

#### Prevention

Prevention was the third most salient word across the three groups. Spanish speakers had a higher Smith’s S (0.10) compared to English speakers (0.02).

Many participants described masking as a mandatory risk mitigation measure, often enforced by their child’s school, along with other behaviors such as hand washing, temperature checks, and symptom screenings.

*Parent: We have to wear masks and they have to. We have to come in, we get- we fill out the paper, we get our temperature check, everybody. And then we take our kids to school. When we go out the door, they can take their mask off. But when we get back to the center, they have to put them back on and then we have the temp check them again before they come in.*

### English-speaking Parent, Age 56

*Interviewer: Yeah, um, you mentioned that they hand out masks still. Is that, like, they have to wear it or they just [inaudible].*

*Parent: No, so if, a child comes up to school, they say, hey, I mean they were handing out masks when it was still mandatory. But now, if you still want to wear a mask, they still hand them out to the students. The students will come in and say, can I have a mask? So they just still give them masks.“*

### English-speaking Parent, Age 35

#### Health

The association between “health” and masking was the fourth most salient among Spanish and English speakers but Spanish speakers had a higher Smith’s S score (0.08 vs. 0.02).

Some parents and children described their primary motivation for masking was to maintain their health and reduce morbidity and mortality.

*Entrevistadora: Okay. Y, ¿puede recordar algunas de las primeras conversaciones que tuvo con ella sobre COVID?*

*Interviewer: Okay. And can you remember some of the first conversations that you had with her about COVID?*

*Madre: Si, ehm. Pues hablamos de que estaba este virus que… que era malo. Que eh, por eso estábamos usando los cubrebocas para que no se contagiara, para que no se enfermara y pues pudiera pasar algo peor, ¿no? La muerte.*

*Parent: Yes. Well, we talked about that there was this virus that… that it was bad. That eh, that is why we were using facemask so that she would not get infected, so she would not get sick and well that something worse could happen, no? Death. “*

#### Spanish-speaking Parent, Age 28

##### **Care**

The word “care” (0.05) had a higher salience among Spanish speakers than English speakers (< 0.02). The opposite was true for the word “necessary” with English speakers compared to Spanish speakers (0.04 vs. < 0.02).

Within the interviews, few parents and children described their motivation for masking to protect others or prevent the spread of COVID-19 to others.

*Madre: Pero si le he, le he explicado que es una enfermedad que te, si alguien la tiene pues es contagiosa y, y que debemos si estamos enfermos debemos de guardar los días en casa para no contagiar a más gente. Y también le digo, “mija, en la escuela trata de, dejarte el cubrebocas. Lávate las manos. Este, tu botella de agua.” A ella van dos ocasiones que le agarran el agua accidentalmente.*

*Parent: But I have, I have explained to her that it is a disease that, if someone has it well it is contagious and, and that if we are sick, we should stay at home a couple of days as to not spread it to other people. And also, I tell her, “Darling, at your school try to keep the, the facemask on. Wash your hands. Like, your water.” There have been two occasions in which they accidentally grab her water.*

#### Spanish-speaking Parent, Age 44

*Parent: Okay, tell me some things that you know about COVID.*

*Child: Well, you like to wear mask because the- if you don’t wear a mask, you’re gonna get someone else sick.*

**English-speaking Parent, Age 56 & English-speaking Child, Age 6**

## Discussion

Overall, based on the most salient freelist words and in the context of the parent-child interviews, parents showed support for masking and masking policies in school settings. Although there were a few terms that showed disapproval (unnecessary, mandatory, can’t breathe), they had lower salience indicating a minority view towards masking in school communities. Parents used these freelisting terms safety and protection, and in the context of interviews, they spoke favorably regarding masking as a risk mitigation strategy for COVID-19 and potentially other airborne diseases. Our findings may suggest that the politicization of masking policy is not a widely held view in marginalized communities that are most vulnerable to airborne diseases like COVID-19.

This population is very specific to San Diego and is not generalizable. However, the diversity of these communities can help inform public health and school officials in different communities. Our findings come from a snapshot in time, but it still provides a rich and important source of data. Additionally, some freelist words did not have direct equivalents between Spanish and English or vice versa, but we believe it is still important to include due to the large Hispanic demographic composition of the communities we were working with. We chose to collect race/ethnicity data on the survey participants’ children rather than on the participants themselves. We, therefore, used language as a proxy for culture.

There were some notable differences between Spanish-speaking and English-speaking participants regarding words associated with masking. The words protection and prevention had a higher salience value among Spanish-speaking participants than English speakers. This could mean that Spanish-speaking participants agree that masking is a form of risk mitigation more so than English speakers. One reason for this more positive view of masking may be that Spanish-speaking communities in San Diego County were hit harder at the beginning of the COVID-19 pandemic [32]. Nationally, immigrants and people of color had higher morbidity and mortality than native-born and white individuals [33]. The one child-participant’s comment about not wanting to die and having lost loved ones to COVID-19 highlights what this community has faced during the pandemic.

There is clear evidence that communities with high risk for COVID-19 exposure are exacerbated by discriminatory socio-economic factors, such as racialization, sexism, and deregulation of workplace safety and social supports [12]. The pandemic did create opportunities to close the health equity gap with increased access to free COVID-19 vaccine and diagnostic testing [34]. However, as of May 2023, though COVID-19 cases and mortality are still high in the United States, federal, state, and local governments have rolled back these supports [35]. Individual-level risk mitigation strategies, such as masking, have become the only tools left for vulnerable communities to protect themselves.

Masking is a relatively cheap effective risk mitigation tool that many school parents and children view favorably. Our findings build on other studies that found masking is generally supported in the general population [36]. Our PCIs showed that our parent participants also were not against mandatory masking at school but felt that it was necessary to protect their children. Public health officials have a responsibility to prioritize the views of those most impacted by diseases like COVID-19 when deciding on risk mitigation policies like school masking. Masking can be a tool to protect communities that have inequitably shouldered the burdens of the COVID-19 pandemic. Assuming masking preference based on the most vocal constituents takes away the agency of the most vulnerable to protect themselves and their loved ones. We also recommend policymakers continue to make free masks available to school communities.

## Conclusion

While masking and masking policy has been effective against COVID-19, their politicization has restricted the voices of vulnerable school communities. Our results have shown that masking perspectives among Spanish-speaking parents and children associate masking with words like safety, prevention, protection, care, and health. Our PCI interviews showcase positive views of masking that undercut assumptions of masking as not a favorable risk mitigation strategy and masking policy as unwanted in communities that are most at risk for COVID-19 spread. Communities at risk are left with masking as their only option. It is an affordable and effective tool that gives agency to individuals in vulnerable communities. We recommend that policymakers continue to offer resources that allow school communities to monitor their risks.

## Data Availability

Study data will be publicly available through the University of California San Diego Library Research Data Curation Repository. https://library.ucsd.edu/research-and-collections/research-data/index.html
